# Restricted Water Intake and Hydration with Fructose-Containing Beverages during Infancy Predispose to Aggravate an Acute Renal Ischemic Insult in Adolescent Rats

**DOI:** 10.1155/2020/4281802

**Published:** 2020-11-04

**Authors:** Fernando E. García-Arroyo, H. Emmanuel Pérez-Estévez, Edilia Tapia, Guillermo Gonzaga, Itzel Muñoz-Jiménez, Virgilia Soto, Horacio Osorio-Alonso, Nayelli Nájera, Eduardo Meaney, Guillermo Ceballos, L. Gabriela Sánchez-Lozada

**Affiliations:** ^1^Department of Cardio-Renal Physiopathology, INC Ignacio Chavez, Mexico City 14080, Mexico; ^2^Sección de Estudios de Posgrado e Investigacion, Escuela Superior de Medicina, Instituto Politécnico Nacional, Mexico City, Mexico; ^3^Department of Pathology, INC Ignacio Chavez, Mexico City 14080, Mexico

## Abstract

We aimed to investigate the effects of chronic fluid restriction and hydration with a sweetened beverage (SB) in rats from weaning until adolescence, in a posterior acute kidney injury (AKI) event induced by ischemia-reperfusion (I/R). We followed 5 groups of weaning rats: control group (C); two groups with 22 h/day fluid restriction, a group hydrated for two hours with water (-W) and a group hydrated with SB; one group receiving SB ad libitum all day (+SB); and one group in which water consumption was increased using a gel diet. The rats that reached adolescence were submitted to I/R. Fluid restriction and/or SB hydration induced mild renal alterations that were significantly accentuated in the -SB group and resulted in worse outcomes after I/R-induced AKI that resulted in a catastrophic fall in creatinine clearance and diffuse acute tubular necrosis. In summary, low tap water intakes, as well as SB intake in infancy, prompt kidney worse outcomes in a later event of AKI during adolescence and both insults magnify kidney damage. Studies on hydration habits in children are recommended to disclose the potentially harmful effects that those behavioral patterns might carry to future renal health.

## 1. Introduction

In many countries, water intake in children and adults does not meet the recommendations of adequate daily fluid intake [1–8]. Taste influences hydration and beverage choice in adults and children [9–11]. Thus, the intake of SB has significantly increased [12], likely replacing tap water intake.

In children and adolescents, there is a high prevalence of inadequate total fluid intake. Such an effect has been associated with low water intake [13–16]. In children, the magnitude of rehydration is significantly affected by the flavor of the available beverage [10]. The hydration state in children has been established as >800 mOsm in urine samples [13, 14, 16], a value previously reported as the upper limit of euhydration [17–19]. Urinary osmolality is a biomarker that reflects fluid intake, and a high value has been associated with the risk of developing chronic kidney disease (CKD) [20]. Dehydration augments the risk for acute kidney injury (AKI) [21] that is enhanced by exercising in the heat [22] and the intake of SB [23].

Experimental studies have shown that recurrent dehydration induces chronic overactivation of the polyol pathway and endogenous production of fructose in the renal cortex as well as the increased and continuous secretion of vasopressin [24, 25]. Such effects lead to chronic tubulointerstitial damage [26]. Interestingly, the knockdown of fructokinase, the first step in fructose metabolism, protected from dehydration-mediated kidney injury and prevented the stimulation of vasopressin secretion despite that dehydrated mice had increased serum osmolality [26]. Moreover, rehydration with a fructose-containing beverage worsened renal damage induced by dehydration [27, 28]. In this context, the blockade of V1a and V2 vasopressin receptors with conivaptan prevented the overactivation of aldose reductase and fructokinase and provided nephroprotection [25], an effect that later was shown to be induced by the specific blockade of V2 receptor [24].

Chronic kidney disease or CKD is a worldwide epidemic [29]. Although many cases of CKD are secondary to diabetes or hypertension, up to ≈10% of the cases result from an unknown cause in developing countries; importantly, the disease affects mainly young adults [30]. Currently, it is accepted that even mild episodes of AKI may evolve to CKD [31], a fact also highlighted in the pediatric population [32]. Multiple AKI episodes also confer cumulative risk for CKD [33].

As has been shown that children fail to accomplish an adequate water intake and have a preference for SB, and such habits might promote renal alterations through the chronic activation of vasopressin and polyol-fructokinase pathways, the present study is aimed at investigating the effects of chronic fluid restriction and hydration with SB in rats from weaning until adolescence, in a posterior AKI event induced by I/R.

## 2. Materials and Methods

All studies were approved by the Institutional Animal Care and Use Committee (Permit No. INC/CICUAL/PIL/001/2019) and were performed in accordance with the *Guide for the Care and Use of Laboratory Animals*, 8th edition, and the Mexican regulation on animal use and care (NOM-062-ZOO-1999).

Weaning male Wistar rats (21 days of age, Research Global Solutions, Mexico City, Mexico) were used for these studies. Rats were followed up for 6 weeks until they reached adolescence. This development period in rats represents humans' age from 6 months to 14-17 years old approximately [34–36].

We followed 5 groups of rats (*n* = 6 each): control group (C); two groups with 22 h/day fluid restriction, one was hydrated for two hours with water (-W) and the other was hydrated with a sweetened beverage (-SB) containing a mixture of glucose (3.5%) and fructose (7.5%), which corresponds to the combination used in some popular soft drink brands [37]; in addition, a group that received SB ad libitum all day (+SB) and a group in which water intake was increased using a gel diet (4 mL of water/gr of food) plus liquid regular water (+W) [38]. The vendor provided 10 rats/week for 3 weeks, and 2 rats were allocated to each group on a weekly basis.

After 6 weeks of follow-up, rats were collected for urine and blood and the next day were submitted to AKI by right uninephrectomy and renal I/R protocol of the remaining kidney accordingly to a previously reported method [39]. The right kidney was sagittal cut and one part fixed in 4% paraformaldehyde while the other was split in the cortex and medulla and snap-frozen in liquid nitrogen until further processing. The rats were again collected the next day for urine and euthanized for collecting blood and the left kidney, from which one-third was used to isolate proximal tubules and the remaining two-thirds were preserved in the same way as the right kidney. Tubular fractions were isolated using methods previously reported [24, 40].

Creatinine was measured in plasma and urine samples (Stanbio Direct Creatinine LiquiColor, Boerne, TX, USA), and creatinine clearance (CrCl) was calculated as a proxy of renal function before and after the ischemia-reperfusion procedure. In pre-AKI samples, plasma copeptin was measured by enzyme-linked immunosorbent analysis (ELISA) (Peninsula Laboratories International, San Carlos, CA, USA), urine osmolality was measured with a freezing point osmometer (Löser Messtechnik, Berlin, Germany), nitrate and nitrite concentrations were evaluated in plasma and urine samples with a colorimetric commercial kit (Cayman Chemical, Ann Arbor, MI, USA; Cat. 780001), and plasma sodium was measured by flame photometry (Instrumentation Laboratory, Bedford, MA, USA). In renal cortex homogenates obtained in pre-AKI and post-AKI renal tissue, lipid peroxidation was evaluated by measuring 4-hydroxynonenal as previously reported [41].

Antibodies against kidney injury molecule 1 (KIM-1, Cat. GTX85067, dilution 1 : 2500), fructokinase (KHK, Cat. GTX109591, dilution 1 : 5000), angiotensin-converting enzyme 2 (ACE2, Cat. GTX101395, dilution 1 : 1500), and angiotensin II (Cat. GTX37789, dilution 1 : 1000) were obtained from GeneTex, Inc., Irvine, CA, USA. Moreover, angiotensin II type 1 receptor (AT1-R, Abcam, Cambridge, UK; Cat. ab124734, dilution 1 : 1500) and neutrophil gelatinase-associated lipocalin (NGAL, Santa Cruz Biotechnology, Santa Cruz, CA, USA; Cat. sc-515876, dilution 1 : 5,000) expressions were evaluated in renal cortex tissue obtained pre-AKI and tumor necrosis factor-alpha (TNF-alpha, Santa Cruz Biotechnology, Santa Cruz, CA, USA; Cat. sc-52746, dilution 1 : 5000) and NGAL expressions were evaluated in proximal tubule fractions obtained post-AKI by western blot using beta-actin (GeneTex, Inc., Irvine, CA, USA; Cat. GTX109639, dilution 1 : 5 000) as a loading control. Western blot analyses were performed in three samples randomly chosen from each group. Western blot data was normalized by the method of single protein detection using as internal loading control a housekeeping protein (beta-actin). Proteins of interest and respective loading controls were run in parallel using the same conditions. The bands were visualized using horseradish peroxidase (HRP) secondary antibodies and Immobilon Crescendo Western HRP Substrate (Merck Millipore, Billerica, MA, USA). Immunoblots were analyzed using Image Studio Lite 5.2 software (LI-COR Biosciences, Lincoln, NB, USA). The suitability of beta-actin as a loading control was confirmed by a similar luminescence signal (by densitometric analysis) in all bands in each western blot, therefore confirming that the expression of beta-actin was stable and unaffected by the experimental conditions and sampling method.

Renal histology was qualitatively evaluated in kidney specimens obtained pre- and post-AKI by a pathologist unaware of the different groups in slides stained with periodic acid-Schiff (PAS, to evaluate inflammatory infiltration) and hematoxylin-eosin (HE, to evaluate acute tubular necrosis (ATN)).

### 2.1. Statistical Analysis

The data is presented as mean ± standard deviation as pre-AKI and post-AKI values. The study group data were compared with one-way analysis of variance (ANOVA) followed by Tukey's multiple comparison test using Prism 8 (GraphPad Software LLC., San Diego, CA, USA). *p* values < 0.05 were considered significant.

## 3. Results

### 3.1. Pre-AKI

**Figure 1:**
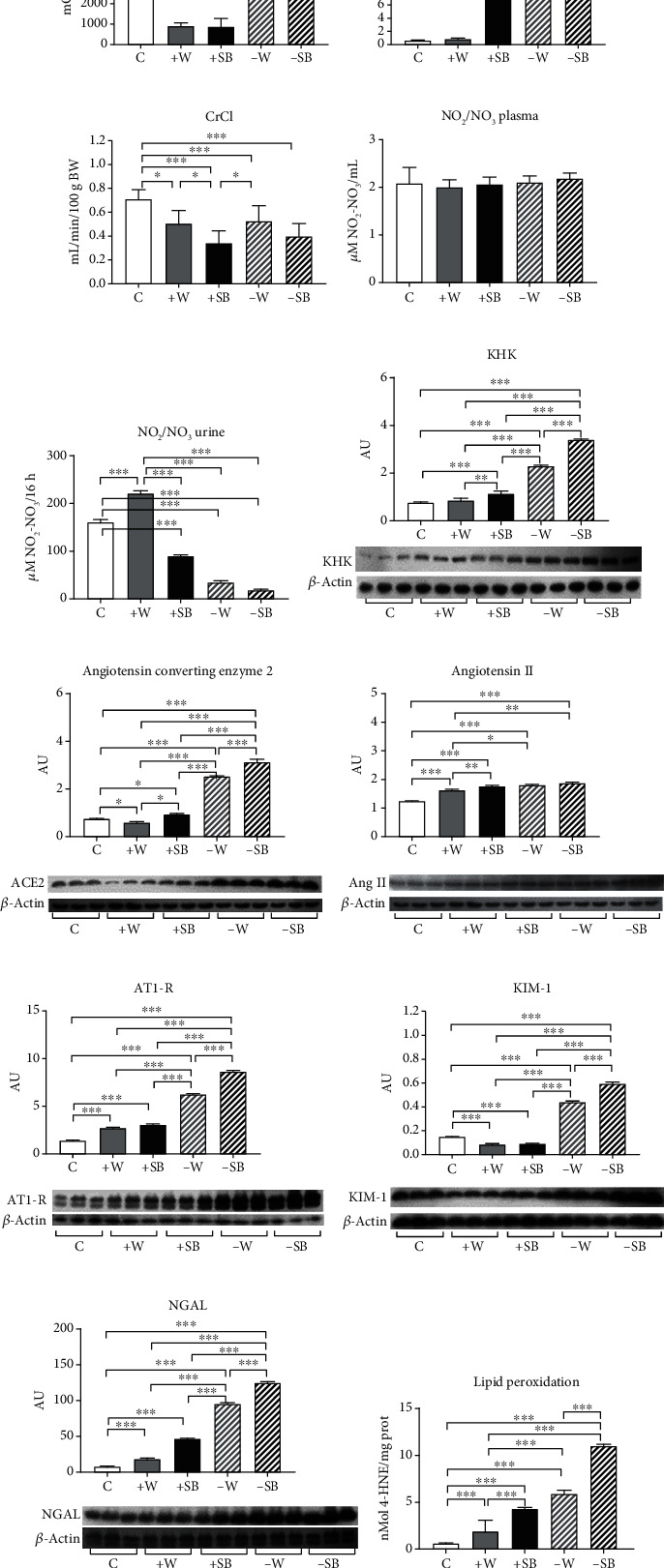
Various parameters at the end of the 6 wks of follow-up evaluated preacute kidney injury (AKI) by ischemia/reperfusion: (a) urinary osmolality; (b) plasma copeptin; (c) creatinine clearance (CrCl); (d) nitrate/nitrite concentration (NO_2_/NO_3_) in plasma; (e) nitrate/nitrite concentration (NO_2_/NO_3_) in urine; (f) fructokinase (KHK) renal cortex expression; (g) angiotensin-converting enzyme 2 renal cortex expression; (h) angiotensin II renal cortex expression; (i) angiotensin II receptor type 1 (AT1-R) renal cortex expression; (j) kidney injury molecule 1 (KIM-1) renal cortex expression; (k) neutrophil gelatinase-associated lipocalin (NGAL) renal cortex expression; (l) lipid peroxidation measured in renal cortex homogenates. C: control; +W: increased water intake; +SB: sweetened beverage (SB) ad libitum; -W: fluid restriction plus hydration with tap water; -SB: fluid restriction plus hydration with SB. ^∗^*p* < 0.05; ^∗∗^*p* < 0.01; ^∗∗∗^*p* < 0.001.

The different hydration states were reflected by the urine osmolality evaluated after 6 weeks of follow-up. Those groups that received increased fluid intake had significantly lower UOsm, and the fluid-restricted ones had significantly higher UOsm (Figure 1(a)). On the other hand, plasma copeptin levels increased due to both fluid restriction and SB intake. The combination of the two stimulators significantly magnified such effect (Figure 1(b)). Plasma sodium mildly but significantly decreased in the +W and +SB groups in comparison to the control (C = 126 ± 12 mEq/dL, +W = 115 ± 4 mEq/dL, and +SB = 110 ± 10 mEq/dL; +W and +SB vs. C *p* < 0.05) while in the fluid-restricted group, the values were not different versus the control (−W = 118 ± 6; −SB = 117 ± 11). Kidney function was estimated as CrCl. All the experimental groups had lower CrCl in comparison to the control (normalized by 100 g/body weight), and such effect tended to be more apparent in both groups that received SB either ad libitum or in fluid-restricted rats (Figure 1(c)). Plasma nitrate/nitrite concentrations (NO_2_/NO_3_) were not different among the groups (Figure 1(d)); however, urinary excretion showed a different pattern: water load increased urine excretion, but SB drank ad libitum significantly reduced NO_2_/NO_3_ urine excretion; fluid restriction further reduced the excretion of nitric oxide (NO) metabolites, and such effect was accentuated in those rats that in addition were hydrated with SB (Figure 1(e)). The right kidney removed before the I/R procedure was evaluated for KHK, ACE, angiotensin II, and AT1-R as well as the markers of tubular damage in KIM-1 and NGAL protein expression. KHK, ACE, Ang II, and AT1-R (Figures 1(f)–1(i)) were increased in overhydrated rats (+W), and such parameters were further overexpressed in fluid-restricted rats, being KHK and AT1-R significantly higher in the -SB group. On the other hand, KIM-1 and NGAL expressions were significantly increased in both fluid-restricted groups, and such effect was magnified by SB hydration (Figures 1(j) and 1(k)). Lipid peroxidation, a marker of oxidative stress, was also significantly increased in all groups in comparison to the control (Figure 1(l)). The combination of fluid restriction plus SB hydration had a synergistic effect; therefore, the -SB group presented the more significant lipid peroxidation (Figure 1(l)). In the histological analysis, there were no morphological changes observed among the different groups (data not shown).

### 3.2. Post-AKI

**Figure 2:**
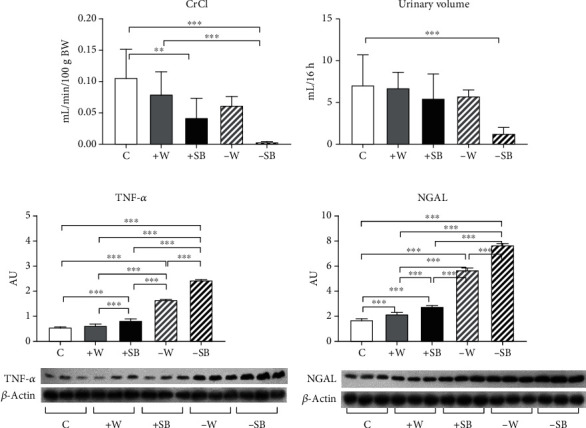
Markers of kidney damage 24 h postacute kidney injury (AKI) by ischemia/reperfusion: (a) creatinine clearance (CrCl); (b) urinary volume; (c) tumor necrosis factor-alpha (TNF-*α*) renal cortex expression; (d) neutrophil gelatinase-associated lipocalin (NGAL) renal proximal tubules expression. C: control; +W: increased water intake; +SB: sweetened beverage (SB) ad libitum; -W: fluid restriction plus hydration with tap water; -SB: fluid restriction plus hydration with SB. ^∗^*p* < 0.05; ^∗∗^*p* < 0.01; ^∗∗∗^*p* < 0.001.

**Figure 3:**
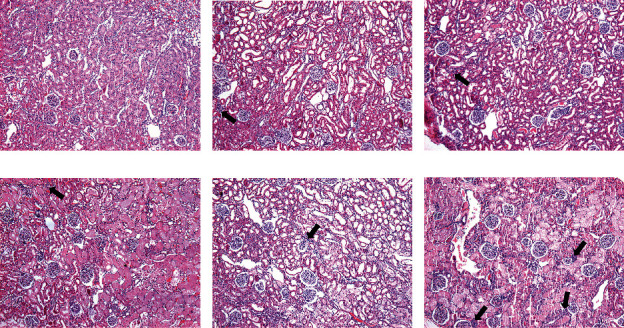
Inflammatory infiltration 24 h postacute kidney injury (AKI) by ischemia/reperfusion. Arrows show areas of inflammatory infiltration. Periodic acid-Schiff-stained sections 100x.

**Figure 4:**
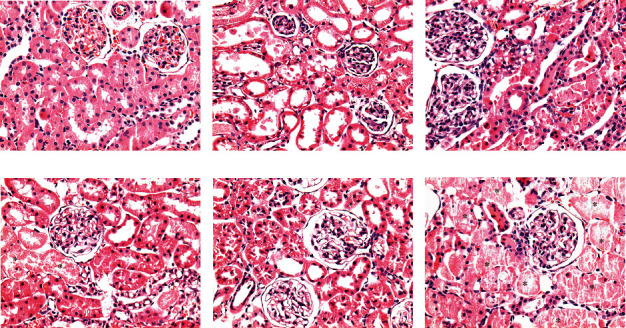
Acute tubular necrosis (ATN) 24 h postacute kidney injury (AKI) by ischemia/reperfusion. Asterisks show areas of ATN. Hematoxylin-eosin-stained sections 400x.

I/R-induced AKI did not produce mortality in the control group. However, it induced mortality of 10-30% in the +W and +SB groups and 50-55% in both groups with fluid restriction. In fluid-restricted groups, AKI had profound effects on kidney function and structure. Fluid-restricted rats hydrated with SB (-SB) were the most affected as CrCl approached zero (Figure 2(a)), and the urine volume was the lowest in this group (Figure 2(b)). By western blot, it was observed that both fluid-restricted groups (-W and -SB) had a high expression of the proinflammatory cytokine TNF-alpha in renal cortex homogenates (Figure 2(c)) and that the effect was significantly exacerbated in the -SB group. NGAL expression was also evaluated in proximal tubule fractions (Figure 2(d)). We found a significant increase in NGAL expression that was better observed in fluid-restricted groups and further accentuated in rats that were hydrated with SB. I/R induced mild ATN and flattening of the tubular epithelium in control rats, and those effects were enhanced in water overload and fluid-restricted rats rehydrated with water (Figure 3). On the other hand, both SB groups had the highest ATN score, and the fluid-restricted group had more inflammatory infiltration (Table 1 and Figures 3 and 4).

## 4. Discussion

Behavioral and environmental factors have been regarded as contributors to the development of chronic diseases. The fluid intake, as a means for preserving an adequate hydration state, is influenced by gender, age, taste, availability, convenience, etc. An inadequate state of hydration is not uncommon as a result of involuntary or voluntary dehydration [42, 43]. Thus, involuntary dehydration is associated with an increased threshold for thirst (aging) and a reduced or lack of tap water intake (unavailability of clean water to drink, etc.) while voluntary dehydration, a condition where individuals do not drink appropriately in the presence of adequate fluid supply, can occur in some types of jobs mainly associated with hot environments and hard labor [44], due to the lack of public toilet facilities, mainly in women [45], due to unpleasant taste in drinking fluids, etc.

It has been shown that voluntary dehydration also occurs in school kids in whom urinary osmolalities greater than 800 mOsm have been reported [13, 17, 19]. Moreover, as taste has a significant influence on hydration preferences in children [10, 11], this fact likely has stimulated the substitution of tap water intake for SB intake. Therefore, both chronic low fluid intake and hydration with SB might have detrimental consequences in renal health making this organ more susceptible to future acute insults.

In the present studies, we found that dehydration induced by restricted fluid intake from weaning until adolescence induced the activation of mechanisms largely known to affect kidney health, such as increased vasopressin secretion, overexpression of components of the renin-angiotensin system, decreased urinary excretion of NO metabolites, increased expression of renal fructokinase, and increased oxidative stress. Activation of such a plethora of mechanisms was associated with a significant fall in CrCl and considerable tubular damage, as suggested by the increased expression of KIM-1 and NGAL. In addition, most of such deleterious effects were enhanced when, in addition, rats were hydrated with SB. Therefore, in the fluid-restricted plus SB intake group, an acute renal damage insult induced by I/R resulted in worse outcomes compared with the fluid-restricted group hydrated with water as CrCl approached to zero; the urinary volume was the lowest; the ATN score was the highest; TNF-alpha and NGAL expressions were the highest as well as oxidative stress. These findings suggest that hydration habits in youth may contribute to accelerated kidney damage and might partly explain why CKD epidemics have burst. Of interest, in a significant proportion of cases, the origin of the disease is unknown [46]. Therefore, these findings could also help to expand the knowledge of the early mechanisms of renal damage.

We also confirmed that SB hydration ad libitum also induced renal alterations [47]. Of note, unrestrained SB induced a significant increase in vasopressin secretion of the same magnitude as fluid restriction and hydration with water, although plasma sodium and urinary osmolality were significantly decreased. Therefore, this finding confirms previous reports in which fructose induced vasopressin secretion through a nonosmotic activation [48, 49] and suggests that despite the high vasopressin secretion, there was an escape of antidiuresis as this group was mildly hyponatremic [50]. Importantly, this group also had worse outcomes after I/R injury in comparison to the control group, therefore pointing out the deleterious effects that SB impose on kidney health as has been suggested in epidemiological studies [51, 52].

On the other hand, overhydration with water also induced mild renal alterations in pre-AKI as this group had a significantly lower CrCl in comparison to the control group. This effect might be partly explained by the slight renal overexpression of angiotensin II and AT1-R receptor likely induced by the mild hyponatremia developed in this group. Despite such effect, this water overhydrated group did not have a worse outcome post-AKI in comparison to the control group.

In summary, these findings suggest that low tap water intakes, as well as SB intake in infancy, prompt kidney worse outcomes in a later event of AKI during adolescence. The combination of both insults synergizes, therefore resulting in a devastating loss of renal function after AKI. Studies on hydration habits in children are recommended to disclose the potentially harmful effects that those behavioral patterns might carry to future renal health.

## Figures and Tables

**Table 1:** Qualitative evaluation of kidney damage post-AKI.

Group	Acute tubular necrosis	Inflammatory infiltration
C	+	+
+W	++	+
+SB	+++	+
-W	++	+
-SB	+++	++

Acute tubular necrosis score: + = mild only in the medulla; ++ = moderate in the medulla and mild in the cortex; +++ = diffuse affecting both the medulla and the cortex. C: control; +W: increased water intake; +SB: sweetened beverage (SB) ad libitum; -W: fluid restriction plus hydration with tap water; -SB: fluid restriction plus hydration with SB.

## Data Availability

The data associated with this study is available from the corresponding author upon request.
